# A physiological perspective on fisheries‐induced evolution

**DOI:** 10.1111/eva.12597

**Published:** 2018-02-16

**Authors:** Jack Hollins, Davide Thambithurai, Barbara Koeck, Amelie Crespel, David M. Bailey, Steven J. Cooke, Jan Lindström, Kevin J. Parsons, Shaun S. Killen

**Affiliations:** ^1^ Institute of Biodiversity, Animal Health and Comparative Medicine University of Glasgow Glasgow UK; ^2^ Fish Ecology and Conservation Physiology Laboratory Department of Biology and Institute of Environmental Science Carleton University Ottawa ON Canada

**Keywords:** anthropogenic change, ecophysiology, fishing, harvest‐induced selection, metabolic rate

## Abstract

There is increasing evidence that intense fishing pressure is not only depleting fish stocks but also causing evolutionary changes to fish populations. In particular, body size and fecundity in wild fish populations may be altered in response to the high and often size‐selective mortality exerted by fisheries. While these effects can have serious consequences for the viability of fish populations, there are also a range of traits not directly related to body size which could also affect susceptibility to capture by fishing gears—and therefore fisheries‐induced evolution (FIE)—but which have to date been ignored. For example, overlooked within the context of FIE is the likelihood that variation in physiological traits could make some individuals within species more vulnerable to capture. Specifically, traits related to energy balance (e.g., metabolic rate), swimming performance (e.g., aerobic scope), neuroendocrinology (e.g., stress responsiveness) and sensory physiology (e.g., visual acuity) are especially likely to influence vulnerability to capture through a variety of mechanisms. Selection on these traits could produce major shifts in the physiological traits within populations in response to fishing pressure that are yet to be considered but which could influence population resource requirements, resilience, species’ distributions and responses to environmental change.

## 1 INTRODUCTION

Commercial and recreational fishing are changing the phenotypic composition of exploited fish stocks, particularly for traits related to life histories and reproduction (Enberg, Jørgensen, Dunlop, Heino, & Dieckmann, 2009; Enberg et al., 2012; Hard et al., 2008; Heino et al., 2013; Jørgensen et al., 2007). Where the high mortality imposed by fishing extends to immature life‐history stages, fishing selects for individuals which reproduce at an earlier age (Ernande, Dieckmann, & Heino, 2004; Heino, 1998; Jørgensen, Ernande, & Fiksen, 2009; Jørgensen, Ernande, Fiksen, & Dieckmann, 2006; Law, 2000; Law & Grey, 1989). Consequently, exploited stocks can become comprised of individuals that mature earlier and at smaller sizes. These effects can become exacerbated when there is direct size‐selectivity by fisheries in which larger fish are preferentially targeted. If the traits under selection by fisheries have a heritable component, then evolutionary change in exploited populations may occur, a phenomenon known as fisheries‐induced evolution (FIE). As evidence of fisheries‐induced evolution has accumulated, focus has shifted from determining whether or not FIE is occurring, to assessing the rate at which these changes occur and the potential for reversibility (Enberg et al., 2012; Heino et al., 2013). It is now recognised that quantifying and predicting these evolutionary responses will be important in maintaining the economic and ecological viability of fisheries (Laugen et al., 2014). This, in turn, requires a thorough understanding of the mechanisms of fish capture, and how traits influence susceptibility to capture for individual fish.

Intraspecific variation in traits related to physiology and behaviour has recently received increased research attention in the wider field of biology (Killen, Calsbeek, & Williams, 2017; Williams, 2008). The study of intraspecific variation in behavioural traits has shown that differences are stable over time and across contexts (Bell, Hankison, & Laskowski, 2009; Wolf & Weissing, 2012), in a diverse array of taxa including fishes (Sih, Bell, & Johnson, 2004). Differences in behaviour among individuals are often correlated with other, more cryptic aspects of an individual's biology, from physiological traits, such as metabolic phenotype (Metcalfe, Van Leeuwen, & Killen, 2016), to whole‐animal measures of performance and fitness (Biro & Stamps, 2008; Careau & Garland, 2012). In the context of fisheries, where these traits influence an individual's susceptibility to capture in a given fishery, and are also heritable (Table 1), harvest‐associated selection can occur and narrow the range of phenotypes within exploited populations (Heino & Godø, 2002). While the role of individual variation in behaviour has been considered in terms of making some fish more vulnerable to capture by fisheries (Biro & Post, 2008; Diaz Pauli & Sih, 2017; Heino, Díaz Pauli, & Dieckmann, 2015; Uusi‐Heikkilä, Wolter, Klefoth, & Arlinghaus, 2008), there has been comparatively little effort to examine how traits other than size at age might make some individual fish more vulnerable to capture than others, particularly the role of physiological traits (Enberg et al., 2012). There have also been few investigations of how increased mortality and altered life histories stemming from harvest may have indirect effects on the physiological traits present within populations (Duffy, Picha, Borski, & Conover, 2013; Jørgensen & Fiksen, 2010; Jørgensen & Holt, 2013).

**Table 1:** Examples heritability estimates for several physiological and behavioural traits potentially related to vulnerability to capture in fish. Where possible, preference for inclusion in table was given to studies using fishes. For several metabolic traits, however, there is a paucity of information for heritability in fish species, and so estimates from other taxa are shown

	Order	Species	Trait	Heritability	References
Physiology	Stylommatophora	*Cornu aspersum*	Standard metabolic rate	0.33	Bruning et al. (2013)
	Orthoptera	*Gryllodes sigillatus*	Resting metabolic rate	0.14	Ketola and Kotiaho (2009)
			Active metabolic rate	0.72	Ketola and Kotiaho (2009)
	Squamata	*Thamnophis sirtalis*	Feeding physiology	0.26	Burghardt, Layne, and Konigsberg (2000)
	Passeriformes	*Ficedula hypoleuca*	Resting metabolic rate	0.43	Bushuev, Kerimov, and Ivankina (2010)
		*Taeniopygia guttata*	Basal metabolic rate	0.45	Mathot, Martin, Kempenaers, and Forstmeier (2013)
	Carnivora	*Cyanistes caeruleus*	Resting metabolic rate	0.59	Nilsson, Akesson, and Nilsson (2009)
		*Mustela nivalis*	Resting metabolic rate	0.54	Szafranska, Zub, and Konarzewski (2007)
	Rodentia	*Mus domesticus*	Basal metabolic rate	0.09	Dohm, Hayes, and Garland (2001)
			Basal metabolic rate	0.38	Konarzewski, Książek, and Łapo (2013)
		*Phyllotis darwini*	Basal metabolic rate	0.21	Bacigalupe, Nespolo, Bustamante, and Bozinovic (2004)
			Maximum metabolic rate	0.69	Nespolo, Bustamante, Bacigalupe, and Bozinovic (2005)
	Cyprinodontiformes	*Heterandria formosa*	Temperature tolerance	0.2	Doyle, Leberg, and Klerks (2011)
		*Poecilia reticulata*	Sensitivity to light	0.36	Endler, Basolo, Glowacki, and Zerr (2001)
	Gasterosteiformes	*Gasterosteus aculeatus*	Burst swimming	0.41	Garenc, Silversides, and Guderley (1998)
	Perciformes	*Dicentarchus labrax*	Stress responsiveness	0.08	Volckaert et al. (2012)
				0.34	Vandeputte et al. (2016)
			Maximum swim speed	0.48	Vandeputte et al. (2016)
		*Oreochromis niloticus*	Temperature tolerance	0.09	Charo‐Karisa, Rezk, Bovenhuis, and Komen (2005)
		*Stegastes partitus*	Swimming stamina	0.21	Johnson, Christie, and Moye (2010)
	Salmoniformes	*Salvelinus fontinalis*	Stress responsiveness	0.6	Crespel, Bernatchez, Garant, and Audet (2011)
		*Salvelinus namaycush*	Depth regulation	0.58	Ihssen and Tait (1974)
		*Salmo salar*	Stress responsiveness	0.23^a^	Fevolden, Roed, Fjalestad, and Stien (1999)
Behaviour	Cyprinodontiformes (fi)	*Poecilia reticulata*	Chase behaviour	0.25	Cole and Endler (2015)
				0.3	Cole and Endler (2015)
				0.03	Cole and Endler (2015)
				0.07	Cole and Endler (2015)
	Cypriniformes	*Danio rerio*	Shoaling	0.4	Wright, Rimmer, Pritchard, Krause, and Butlin (2003)
			Boldness	0.76	Ariyomo, Carter, and Watt (2013)
				0.36	Ariyomo et al. (2013)
	Perciformes	*Archocentrus siquia*	Boldness	0.37	Mazué, Dechaume‐Moncharmont, and Godin (2015)
			Exploration	0.3	Mazue et al. (2001)
		*Megalopyge opercularis*	Escape	0.9	Gervai and Csányi (1985)
			Swimming	0.84	Gervai and Csányi (1985)
			Creeping	0.85	Gervai and Csányi (1985)
			Floating	0.13	Gervai and Csányi (1985)
			Air gulping	0.94	Gervai and Csányi (1985)
	Salmoniformes	*Oncorhynchus kisutch*	Spawning date	0.44	Neira et al. (2006)
		*Salmo trutta*	Boldness	0.01	Kortet, Vainikka, Janhunen, Piironen, and Hyvärinen (2014)
			Freezing	0.14	Kortet et al. (2014)

a Mean of four heritability estimates across four age groups.

Physiological traits related to bioenergetics and swim performance are especially likely to affect the probability that a fish will be captured by fishing gear or survive after escape. For example, minimum metabolic rate (i.e., standard metabolic rate in ectotherms, SMR) is a heritable trait that shows wide, repeatable intraspecific variation (Burton, Killen, Armstrong, & Metcalfe, 2011; Rønning, Jensen, Moe, & Bech, 2007). SMR influences demand for food and oxygen and is related to various aspects of foraging and predator avoidance (Killen, Marras, & McKenzie, 2011; Killen, Marras, Ryan, Domenici, & McKenzie, 2012; Millidine, Armstrong, & Metcalfe, 2006), which could include avoidance of fishing gear. This estimate of basal energetic demand is sometimes also referred to as resting metabolic rate (RMR). Aerobic scope (AS) is the difference between maximum metabolic rate (MMR) and SMR and is the capacity to supply oxygen for aerobic metabolism above that required for maintenance. It sets the limit for aerobic processes that can be performed simultaneously (e.g., activity, growth, digestion) and may affect various aspects of behavioural ecology and the geographical distribution of species (Jørgensen et al., 2012; Killen, Calsbeek et al., 2017; Killen, Marras, Nadler, & Domenici, 2017; Killen, Marras, Steffensen, & McKenzie, 2012; Marras et al., 2015; Pörtner & Farrell, 2008). In fishes, AS is also correlated with swimming endurance, maximum sustainable speed and recovery rate after exhaustive exercise (Killen, Marras, Steffenson et al., 2012; Marras, Claireaux, McKenzie, & Nelson, 2010), all of which may be relevant to a fish's ability to evade capture by fishing. After controlling for factors such as body size and temperature, it is common for metabolic rates to differ by twofold to threefold among individuals of the same species (Burton et al., 2011; Norin & Malte, 2011, 2012). There is also evidence that metabolic rates are at least partially heritable (Table 1) and so could be targets for harvest‐associated selection (Ward et al., 2016).

Variation in sensory ability, neuroendocrinology and cognition among individual fish may also influence fish vulnerability to capture. For example, intraspecific variation in the visual capabilities of fish (e.g., opsin expression in the retina, (Fuller, Carleton, Fadool, Spady, & Travis, 2005; Flamarique, Cheng, Bergstrom, & Reimchen, 2013)) can manifest as differences in how individuals perceive colour, identify shapes and distinguish objects. In addition to the role vision plays in determining whether a gear is perceived by a fish, these traits may also play a affect whether fish adopt specific behaviours upon encountering a gear (Kim & Wardle, 2003). Chemosensory ability (e.g., expression of receptor proteins within the olfactory bulb, or the relative size of the telencephalon or bulb itself) and circulating hormone levels (e.g., ghrelin, a regulator of appetite) may also influence the ability to detect or encounter deployed gears. Broad measures of sensory physiology (e.g., brain size and morphology) show intraspecific variation (Kihslinger, Lema, & Nevitt, 2006) and have also been found to correlate with the likelihood of a fish expressing behaviours potentially related to capture vulnerability (Burns & Rodd, 2008; Wilson & McLaughlin, 2010), as well as cognitive capacity and aspects of decision‐making in fish (Burns & Rodd, 2008). These latter traits relate to an individual's capacity to locate potential escape routes in a trawl, or the entry to a trap (Klefoth, Skov, Kuparinen, & Arlinghaus, 2017; Monk & Arlinghaus, 2017). While circulating levels of hormones and the expression of receptor proteins within the olfactory bulb also play important roles in determining whether certain behaviours are adopted, these are often linked to physiological condition (Hoskins, Xu, & Volkoff, 2008; Volkoff, Xu, MacDonald, & Hoskins, 2009). In these cases, circulating hormone levels may influence energy demand and vulnerability to capture, but may not directly alter susceptibility in their own right.

Fishing may well be causing unnoticed changes to the intrinsic physiological traits of fish which could in turn be influencing species’ life‐history traits, geographical distributions and capacity to respond to environmental change or recover from overexploitation. The current failure to consider these underlying physiological mechanisms in the context of FIE also precludes the development of effective mitigation strategies that refine harvest techniques or gear implementation to better understand the effects of harvest‐induced selection. In this paper, we discuss this gap in knowledge and suggest how studying the role of physiology in FIE from several perspectives will help us understand how selection occurs at the interface between individual fish and fishing gear and how physiological traits could determine which fish are captured and which are not.

## 2 THE CAPTURE PROCESS AND SELECTION ON PHYSIOLOGICAL TRAITS

**Figure 1:**
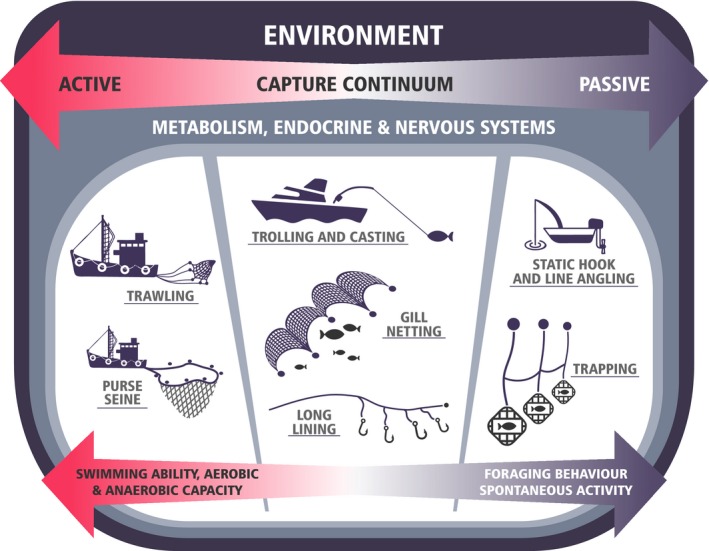
The continuum of fisheries harvest techniques between active and passive gears and practices. Techniques towards the passive end of this continuum are more likely to select on traits associated with foraging behaviour, including hormonal regulation of hunger and exploratory behaviours, as well as sensory ability. Techniques towards the active end of the continuum are more likely to select on traits related to locomotor and escape ability. Broadly spanning the entire continuum are physiological traits related to whole‐animal metabolic traits, which can be directly or indirectly linked to foraging, body size, and locomotor ability. The environment will also have an over‐riding influence along all points of the continuum, modulating fish vulnerability to capture and the strength of potential links with physiological traits

There is great diversity in fishing gears used around the globe by the commercial and recreational fishing sectors spanning marine and freshwater systems. Fishing gears are often divided into passive or active gears. In reality, however, many fishing gears lie along a continuum between these two extremes (Figure 1) and rely on a mixture of stimuli that elicit various behavioural and physiological responses in fish that facilitate their capture. At one end of this continuum, passive gears depend on fish to find the deployed gear, depending on fish foraging behaviour and associated physiological traits. This would include metabolic demand and hormonal cues underlying foraging motivation and also sensory systems related to detecting and finding food sources (e.g., sight and olfactory systems). At the other end of the continuum, active gears pursue or target fish, with vulnerability potentially depending on fish escape ability. This could relate to a range of physiological traits associated with locomotor ability as well as threat detection and evasion (e.g., auditory and visual cues). Differences in capture methods may therefore give rise to differences in selectivity and how that selectivity may be mitigated.

**Figure 2:**
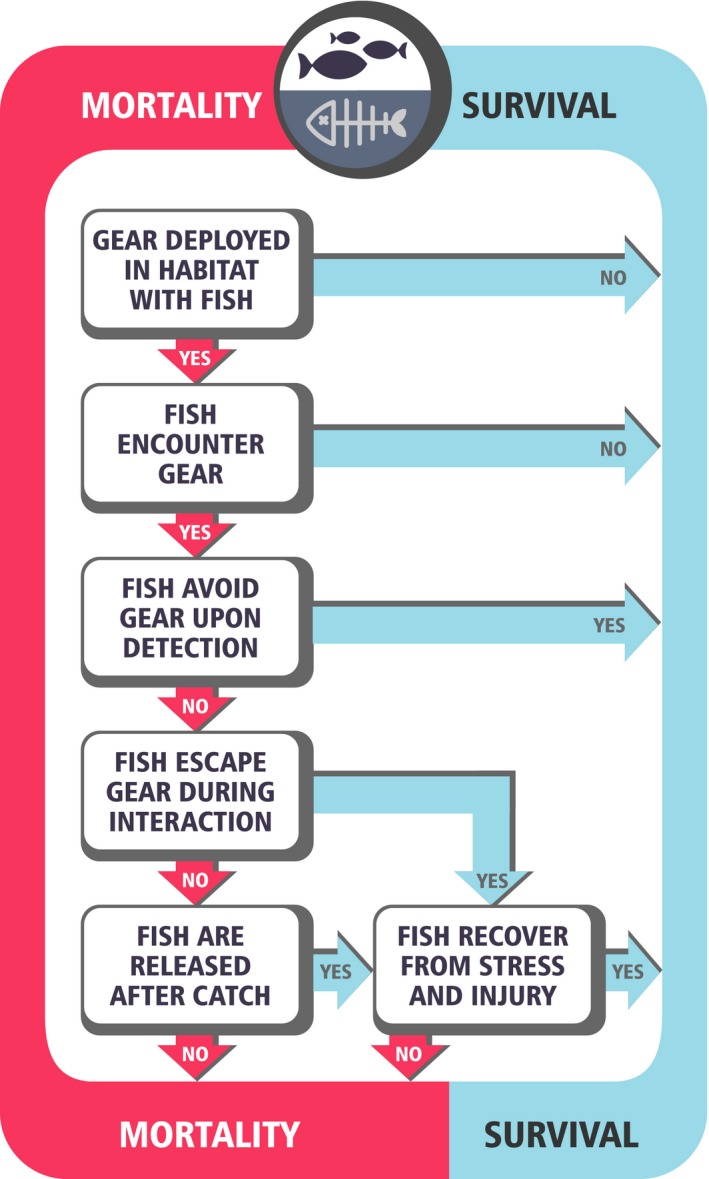
Stages during fishing leading to mortality or survival for targeted fish. Physiological traits are likely to play a role in determining the path taken at each decision point

The capture success for any type of gear is determined by the cumulative probability of outcomes along a set sequence of decision points (Figure 2; Rudstam, Magnuson, & Tonn, 1984; Sampson, 2014). Each of these stages is associated with a specific mechanism of selectivity occurring at different spatial and temporal scales (Millar & Fryer, 1999) that may act together to determine an individual's overall vulnerability to capture. The outcome at each stage has the potential to be influenced by the physiological traits of individual fish. In addition, the environment will have a profound effect on fish physiology, possibly modulating the outcomes and the degree of selectivity at each stage.

### 2.1 Selection via habitat use

The broadest spatial scale of selection occurs with the initial deployment of the gear: only those individuals within the active space of a given gear will be available to the fishery. Within‐species differences in habitat use have been observed in fishes (Elliott, Turrell, Heath, & Bailey, 2017; Kobler, Klefoth, & Arlinghaus, 2008) and can vary based on size, sex or reproductive stage (Sólmundsson et al., 2015). The role of physiological traits in determining habitat use in fishes has received little attention so far but are very likely to affect capture vulnerability at broad spatial scales because parameters that affect gear deployment such as depth distance from shore, and sea bed types are linked to variation in food abundance, predation risk, temperature, oxygenation and water velocity. SMR can influence food and oxygen requirements in fish (Killen, Marras, Ryan et al., 2012; Killen et al., 2011), and both SMR and AS are strongly dependent on temperature (Biro & Stamps, 2010; Claireaux & Lefrancois, 2007; Fry, 1971). These temperature effects on metabolic physiology could in turn influence habitat use by individual fish and their likelihood of encountering deployed gears. For example, thermal variation drives seasonal southward migrations of Pacific bluefin tuna (*Thunnus orientalis*; Whitlock et al., 2015), and diurnal patterns of habitat use in small spotted catsharks (*Scyliorhinus canicula*; Sims et al., 2006), as individuals seek to avoid energetically costly hunting grounds, and maximise the efficiency of feeding and subsequent digestion. Variation in temperature preference and habitat use may also be directly linked to individual SMR (Table 1) and the maximisation of aerobic scope and capacity for growth (Killen, 2014). Similarly, fish often use specific habitats for certain behaviours, such as foraging (Bernal, Brill, Dickson, & Shiels, 2017; Bernal, Sepulveda, Musyl, & Brill, 2009; Schaefer, Fuller, & Block, 2007), and so individual differences in the frequency or duration of those behaviours may contribute to intraspecific differences in habitat use. Individual boldness, a trait which can correlate with SMR (Huntingford et al., 2010; Killen et al., 2011; Killen, Marras, Ryan et al., 2012), may also affect microhabitat use, with shier individuals associating with shelter. In cases where gears are deployed near available shelters, this could cause shy individuals within a population to be more likely to be captured (Wilson, Binder, McGrath, Cooke, & Godin, 2011) with correlated selection on metabolic traits.

Physiological traits may also determine the extent to which individual fish exploit vertical habitats. In the open ocean, fish often experience cold, hypoxic conditions during oscillatory dives which approach or extend beyond the depths at which the mixed layer ends and the oxygen minimum zone begins (Bernal et al., 2009). Segregation of vertical habitat use of open ocean fishes is driven by the physiological ability of species to maintain sufficient cardiorespiratory capacity for active behaviours under these challenging conditions (Bernal et al., 2009, 2017), a trait governed by a suite of physiological factors which are known to show interindividual variation (Joyce et al., 2016; Ollivier, Marchant, Le Bayon, Servili, & Claireaux, 2015; Ozolina, Shiels, Ollivier, & Claireaux, 2016). This variation may ultimately manifest as differences in the maximum depth attainable by individual fish, or the amount of time fish spend at a given depth, and so give rise to intraspecific differences in vertical habitat use (Cosgrove, Arregui, Arrizabalaga, Goni, & Sheridan, 2014; Quayle, Righton, Hetherington, & Pickett, 2009; Vaudo et al., 2014), with implications for whether individuals are available to gears deployed at specific depths (Olsen, Heupel, Simpfendorfer, & Moland, 2012). Links between physiological traits and habitat use may also occur indirectly. For example, recent research on pumpkinseed sunfish captured from littoral and limnetic habitats revealed divergent sensitivity of the hypothalamic–pituitary–interrenal axis to stressors (Belanger, Peiman, Vera‐Chang, Moon, & Cooke, 2017) emphasizing potential for spatially structured fisheries to select for stress responsiveness (Table 1).

### 2.2 Selection via gear encounter rate

Although gear encounter rate will necessarily have some overlap with traits affecting habitat selection, there is an important distinction to be made between selective processes occurring at these two scales. Even if gear and fish co‐occur in the same broad habitat, fish must actually encounter the gear to have any chance at being captured. Individual fish vary in spontaneous activity, boldness and exploration (Table 1), and those that are more active will have higher encounter rates with fishing gears (Biro & Post, 2008; Uusi‐Heikkilä et al., 2008). This could be due to random encounters during exploration or, particularly when considering passive gears, due to directed movements towards the deployed gear after initial detection. Indeed, bold or more active phenotypes are often associated with higher vulnerability to capture by angling or gill netting (Biro & Post, 2008; but see Cooke, Wilson, Elvidge, & Cooke, 2017; Härkönen, Hyvärinen, Paappanen, & Vainikka, 2014; Kekäläinen, Podgorniak, Puolakka, Hyvärinen, & Vainikka, 2014; Klefoth, Pieterek, & Arlinghaus, 2013; Klefoth et al., 2017; Wilson et al., 2011), but these mechanisms are also likely to increase the probability of a fish being in the path of an oncoming trawl, or beneath a towed lure.

Importantly, the drivers of these behavioural differences may be linked with underlying physiological traits, at least in some contexts (Biro & Stamps, 2010; Killen, Marras, Metcalfe, McKenzie, & Domenici, 2013). Fish that are more active and exploratory, for example, have also been shown to have lower hypothalamus–interrenal–pituitary and parasympathetic reactivity (Øverli, Sørensen, & Nilsson, 2006; Verbeek, Iwamoto, & Murakami, 2008), increased sympathetic reactivity (Verbeek et al., 2008) and increased metabolic rates (Killen et al., 2011). More active fish may also possess an increased AS to accommodate this active lifestyle (Killen, Atkinson, & Glazier, 2010; Killen, Marras, Steffensen et al., 2012), suggesting a mechanism by which passive gears may preferentially capture individuals with a high aerobic capacity. A high AS may also permit prolonged or more active bouts of swimming behaviour in fish (Table 1), or be associated with increased maintenance requirements and foraging demands (Auer, Salin, Anderson, & Metcalfe, 2015; Killen, Glazier et al., 2016), thus increasing their likelihood of encountering gears (Redpath et al., 2010). Such mechanisms may partly explain why largemouth bass (*Micropterus salmoides)* bred for high vulnerability to angling also exhibited higher AS (Redpath et al., 2010). For active gears, encounter rate will be largely dependent on the movement of the gear by fishers, but sonar location can direct boats towards shoals or schools of fish. Gregarious individuals could thus be more likely to be targeted by trawls (Nelson, Soulé, Ryman, & Utter, 1987), producing selection against any metabolic or endocrine traits that promote social behaviour (Killen, Fu, Wu, Wang, & Fu, 2016).

### 2.3 Selection via gear avoidance

Fish still have the opportunity to avoid gears after an initial encounter or detection. For passive gears, traps catch only a proportion of fish that come within a close proximity because some fish enter traps more readily than others (Diaz Pauli, Wiech, Heino, & Utne‐Palm, 2015; Thomsen, Humborstad, & Furevik, 2010). This could depend on a number of physiological factors, including physiological traits that underlie decision‐making and risk assessment (Andersen, Jørgensen, Eliassen, & Giske, 2016; Giske et al., 2013; Höglund et al., 2005; Øverli, Pottinger, Carrick, Øverli, & Winberg, 2002; Øverli, Winberg, & Pottinger, 2005; Winberg & Thörnqvist, 2016; Table 1). For passive gears, it has been suggested that decision‐making after the initial gear encounter is a greater determinant of individual vulnerability to capture than encounter rate itself (Klefoth et al., 2017; Monk & Arlinghaus, 2017). In largemouth bass, individuals with low stress responsiveness are more vulnerable to capture by angling, although the exact stage of the capture process that is affected by endocrine traits was not identified (Louison, Adhikari, Stein, & Suski, 2017). Interestingly, however, boldness and metabolic traits did not influence capture vulnerability in this study, providing evidence that the decision to engage with the deployed gear after discovery was at least partially detached from foraging requirements, exploration or risk‐taking per se. Still, hunger increases willingness to approach and enter baited traps after an initial encounter, as does environmental temperature (Thomsen et al., 2010). While appetite is inherently labile, a higher metabolic rate (Killen et al., 2011) could increase the probability of a fish being hungry and responsive to baits. However, effects of the environment, such as temperature, are often observed on the vulnerability of fish via the cumulative effects of increased activity and feeding motivation (Stehfest, Lyle, & Semmens, 2015; Stoner, 2004). By allowing the expression of extreme phenotypes, this plasticity could weaken selection on heritable traits underlying fish vulnerability to capture, although the capacity to exhibit plasticity itself could be targeted by selection.

Although individuals with enhanced sensory capacity would presumably be better able to avoid certain gears or find baited hooks (Lennox et al., 2017), we are unaware of empirical work directly examining this possibility. For active gears, fish often flee oncoming active gears upon first visual or auditory detection (Handegard & Tjøstheim, 2005). However, there is variation in reaction distance, speed (Table 1) and directionality of this response among individuals that may be related to various aspects of sensory physiology (Winger, Walsh, He, & Brown, 2004). Many gear types encourage high densities of fish within their immediate vicinity, potentially exaggerating the importance of social interactions and associated physiological traits in determining responses to gear (Winger, Eayrs, & Glass, 2010). Should high densities of fish be present around a gear already, asocial fish may be dissuaded from approaching, reducing their capture vulnerability and indirectly selecting on underlying physiological traits related to sociability (Killen, Fu et al., 2016).

Avoidance of active gears also shows interesting parallels with optimal escape theory for avoidance of natural pursuit predators—fleeing too early can result in lost foraging opportunities while fleeing too late can result in mortality (Winger et al., 2010; Ydenberg & Dill, 1986). The costs of lost foraging opportunities may be greater for individuals with higher SMR, causing them to have shorter reaction distance and increased risk of fishing mortality (Finstad, Forseth, Ugedal, & Naesje, 2007; Killen et al., 2011). Individuals with a higher foraging demand may be more likely to perceive the benefit of investigating a food source as outweighing the risk posed by foreign objects such as hooks or traps. Metabolic traits of fish have been shown to correlate with boldness (Huntingford et al., 2010; Killen et al., 2011; Killen, Marras, Ryan et al., 2012), which in turn has been shown to correlate with susceptibility to capture in passive gears (Biro & Post, 2008; Diaz Pauli et al., 2015; Klefoth et al., 2017). It has also been demonstrated that cardiac output and RMR are directly correlated with vulnerability to capture in largemouth bass (Cooke, Suski, Ostrand, Wahl, & Philipp, 2007; Redpath et al., 2010). Such links among traits may partly explain the presence of “timidity syndromes” (Arlinghaus et al., 2017), where bolder individuals are apparently harvested from populations more frequently owing to their increased vulnerability to capture. This is corroborated by observations that fish populations subjected to recreational fishing pressure also exhibit lower RMR than populations with no fishing pressure (Hessenauer et al., 2015).

### 2.4 Selection via escape from gear

Even when capture seems inevitable, fish often escape gears by employing behaviours that are likely linked to aerobic and anaerobic capacity. This is particularly true for active gears such as trawls, which herd fish as they attempt to swim and hold station in front of the trawl mouth. They eventually fatigue, fall back further into the net and finally into the codend, where they are retained (Winger et al., 2010). There may also be a behavioural decision‐making component to this form of capture whereby a fish “voluntarily” ceases swimming before complete exhaustion or follow shoal mates into the trawl. During the final moments of the trawl, free‐swimming fish within the body of the net may also be retained as the gear is hauled to the surface, and accelerates as it lifts from the sea floor. Fish “scooped up” in this fashion do not have to have succumbed to fatigue to be caught. Fish can escape by swimming faster than the trawl (Table 1), or moving around the outside of the trawl mouth, making it highly likely that faster swimming fish, or those with a greater capacity for short‐bursts of anaerobic swimming, escape capture. This effect has been illustrated in laboratory‐based trawling simulations with a direct positive correlation between the capacity for burst‐type anaerobic swimming and the ability to avoid being captured (Killen, Nati, & Suski, 2015). Fish can also escape capture once inside the trawl net by passing through the mesh. Escape through mesh is size‐selective, but there may well be a large influence of swimming endurance and anaerobic capacity on escape ability at this stage (Winger et al., 2010), because fish will require bursts of anaerobic swimming while changing their vector relative to the path of the trawl.

It is unknown whether the metabolic costs of prior feeding and digestion affect vulnerability to capture via reductions in swimming performance. In fish, there is a temporary postfeeding increase in metabolic rate associated with digestion and nutrient assimilation (Fu, Cao, Peng, & Wang, 2008; Jobling, 1995; Secor, 2009), referred to as specific dynamic action, that can reduce swim performance (Alsop & Wood, 1997) and possibly decrease the ability to outswim a trawl. Fish with a larger AS (Table 1), however, might maintain an excess capacity for swimming even while processing a meal, providing another means by which individuals with a higher AS may be less catchable by trawl.

The relevance of this swim performance‐based mode of selectivity is dependent on fish engaging in an optomotor response (a reflexive behaviour thought to reorient a swimming fish after displacement from its desired horizontal course; Kim & Wardle, 2003) in which fish swim to maintain station with the trawl, often oriented adjacent to the trawl doors, until they drop back within the net. While this is often observed (Kim & Wardle, 2003; Rose, 1995), individual variation in the response to gears is also frequent (Underwood, Winger, Fernö, & Engås, 2015; Yanase, Eayrs, & Arimoto, 2009) and often predicts the chances of capture for individual fish. Whether these behavioural responses correlate with heritable physiological traits remains unclear, although individual's orientation prior interaction with the trawl gear, and density of conspecifics, can influence fish responses to trawls (Rose, 1995; Underwood et al., 2015). This would suggest that responses to trawls are influenced by external environment, which could dampen selection on traits correlated with swim performance. Kim and Wardle (2003) noted that erratic responses, characterised by burst swimming and haphazard, rapid changes in orientation, acceleration and swim velocity, lead to opportunistic use of potential escape routes around trawls. Fish with greater anaerobic capacity may be expected to engage in such behaviours more readily, or for longer periods, and so be less catchable.

The physiological traits potentially underlying escape from fishing gears can be highly plastic in response to environmental factors. For example, reduced temperature can decrease swimming ability, aerobic metabolic capacity, reaction distances and fish responsiveness (Claireaux, Couturier, & Groison, 2006; Killen, Nati et al., 2015; Killen, Reid, Marras, & Domenici, 2015; Winger et al., 2004, 2010). Therefore, depending on the temperature, or the season, individual fish may be more or less likely to be captured by gears in a manner directly related to their sensitivity to thermal shifts and corresponding effect on performance. Gears may therefore have a reduced selective impact at cold temperatures, when a higher proportion of the population cannot escape. A similar mechanism could occur at the higher end of the thermal range within species, where some individuals will begin to exhibit decreased performance due to warming. Harvest‐associated selection is therefore likely to be highly dependent on how the environment modulates links between physiological traits and the escape ability of individual fish. Finally, there is wide intraspecific variation in stress responsiveness within fish species (Höglund et al., 2005; Pankhurst, 2011), and so individuals may vary widely in the extent to which they can recover from fishing‐stress and physical trauma even after they escape from a fishing gear (Table 1). Outswimming a trawl may result in a severe physiological disturbance due to intense exercise, as may fighting on a fishing rod or longline. Even relatively benign gears like traps can induce a stress response from confinement (Colotelo et al., 2013). During recovery from these stressors, fish may be more vulnerable to predation or less likely to forage or participate in reproductive activities (Winberg & Thörnqvist, 2016). There is some evidence that increased AS may facilitate faster recovery from acute stress (Killen et al., 2014), but much more information is needed in this area. Overall, mortality occurring in fish postescape from fishing gears could constitute another potential avenue for selection on physiological traits to occur.

## 3 CONSEQUENCES OF SELECTION ON PHYSIOLOGICAL TRAITS BY FISHERIES

### 3.1 Understanding the mechanisms and extent of FIE

As long as we are without a greater understanding of the role of individual physiological traits in selective processes, we will lack a mechanistic understanding of how FIE actually works. Is body size the primary determinant of which fish get captured and which do not, or are there more cryptic underlying physiological factors that are related to these effects? Does the majority of selection and evolutionary change occur in response to capture mechanisms, or are there more nuanced effects stemming from increased mortality with effects on behaviour and physiological traits? (Duffy et al., 2013; Jørgensen & Fiksen, 2010; Jørgensen & Holt, 2013). Even in cases where body size or behaviour are the direct targets of harvest selection, correlated selection on physiological traits would have a range of complex feedbacks and implications for life histories. Traits directly selected upon are often correlated (sharing genetic or phenotypic covariance) with a suite of interrelated physiological, behavioural, morphological and life‐history characters (Salinas et al., 2012). For example, energy allocation within fish is inherently linked to an individual's metabolic traits, as SMR will determine the amount of acquired resources available for investment in physiological functions beyond basic maintenance. Differences among individuals in surplus energy may manifest as differences in morphology, body size and performance, which are thought to targets of harvest‐associated selection (Enberg et al., 2012). For example, increase in liver or reproductive organ size as a result of increased energy storage, or reproductive investment (Blanchard, Druart, & Kestemont, 2005; Craig, MacKenzie, Jones, & Gatlin, 2000; Dahle, Taranger, Karlsen, Kjesbu, & Norberg, 2003; Galloway & Munkittrick, 2006; Hurst, Spencer, Sogard, & Stoner, 2005) can increase fish width relative to its length, increasing its likelihood of retention in the mesh of trawls and gillnets (Enberg et al., 2012). These changes in fish morphology can also be accompanied with reductions in swim performance (Ghalambor, Reznick, & Walker, 2004), with further implications for capture in active gears (Enberg et al., 2012). By considering multiple covarying traits, a more accurate picture of the potential response of the population to selection can be drawn, in instances where body morphology is a direct target of harvest‐associated selection, but correlated selection on metabolic rate and growth capacity (Álvarez & Nicieza, 2005; Burton et al., 2011) could also occur. This could contribute to altered life‐history traits at the population level, with the genetic architecture of related traits (e.g., pleiotropy) possibly influencing the rate and direction of the evolutionary changes.

In addition, increased physiological knowledge of the specific traits targeted by harvest‐associated selection will shed light on the relative roles of plasticity and genetic change in the phenotypic shifts associated with FIE, particularly if the physiological traits that influence vulnerability have a heritable component (Table 1). Evidence of “timidity syndromes” (Arlinghaus et al., 2017) arising in response to fishing pressure has been recorded in recreational fisheries (Januchowski‐Hartley, Graham, Cinner, & Russ, 2015; Twardek et al., 2017). It has so far not been possible to determine whether such phenotypic shifts are due to learned responses, behavioural plasticity, density‐dependent effects or whether they represent evolutionary change. Twardek et al. (2017) revealed that nesting largemouth bass in a long‐term (70 + year) recreational fishing sanctuary provided more attentive and vigorous parental care than fish outside the sanctuary. While this result could reflect at least some component of evolutionary change, additional common‐garden experiments are required to disentangle the confounding environmental effects and phenotypic plasticity.

A greater understanding of whether physiological traits are targeted and selected by the capture process over generations will also aid in identifying the heritable component of the traits that have been observed to shift. If the traits under selection are not heritable, the phenotypic shifts observed are more likely to be the result of plasticity (van Wijk et al., 2013) which can favour a faster recovery in case of the removal of the harvesting pressure. So far, there is no information regarding the genetic basis and architecture of vulnerability to capture and the susceptible genotypes that could be selected against by fishing. Investigating the heritability of the vulnerability to capture itself as well as how this can be genetically correlated with other physiological traits (using quantitative genetics) and genotypes (using genomics) could give critical information in this regard. While phenotypic shifts might be related to plastic responses, it is important to note that plasticity itself often possesses heritable genetic variation that could allow different evolutionary trajectories through genotype‐by‐environment interactions (Nussey, Wilson, & Brommer, 2007; Parsons et al., 2016). Investigating vulnerability to capture, its genetic basis and correlation with other physiological traits across different environmental conditions would then be important to fully understand the mechanisms driving FIE. Changes to population density due to fishing could also be an environmental variable that could generate a new adaptive landscape in which evolutionary changes to behaviour and physiology could occur indirectly. Determining the influence of plasticity in FIE is challenging but studying the role of physiology in selective processes will be key in gaining a full appreciation of these effects.

### 3.2 Understanding environmental modulation of FIE and responses to environmental change

The interplay between fish physiology and FIE will also have complex interactions with environmental factors. Factors such as temperature and oxygen availability have strong effects on fish physiology (Claireaux & Lefrancois, 2007; Fry, 1971). As a result, certain physiological traits could become more or less important for capture vulnerability depending on environmental conditions (Killen et al., 2013). In addition, individuals vary in sensitivity to factors such as temperature, oxygen availability and food deprivation (Biro, Beckmann, & Stamps, 2010; Killen, Marras, Rayan et al., 2012; Killen et al., 2013), and this variation appears directly related to metabolic traits and locomotor ability (Killen et al., 2011; Killen, Marras, Steffensen et al., 2012; Killen, Marras, Ryan et al., 2012; Killen et al., 2013). In the context of fisheries, these effects may cause individual vulnerability to fluctuate across environments if the across‐context repeatability of vulnerability to capture is low (Killen, Adriaenssens, Marras, Claireaux, & Cooke, 2016). In other words, particular individuals that are vulnerable to capture under one set of conditions may be completely different to those most vulnerable under another environment due to individual variation in the physiological reaction norms to changing environmental conditions.

Phenotypic plasticity in response to environmental factors may also alter the degree of variation in vulnerability among individual fish within a population and potentially links between susceptibility to capture and physiological traits (see Figure 1 in Killen et al., 2013). If an environmental condition reduces the variability of phenotypic traits, for example by homogenising the response within a population, the degree of selection of the fishing gears will then be reduced. In addition, if correlations between vulnerability and physiological traits change across environments, due to changes in the variability of either vulnerability or the physiological trait of interest, then the degree of correlated selection on physiological traits due to harvest will change across environments. For example, exposure to hypoxia can cause links between activity and individual metabolic demand that are not observable under normoxia (Killen, Marras, Ryan et al., 2012), stemming from changes in trait variability across environments. Thus, passive fishing gears deployed in hypoxic zones may be more likely to cause correlated selection on metabolic traits.

Such genotype‐by‐environment interactions could have important repercussions on the rate and direction of FIE. Depending on the environment, the degree of selection on the physiological traits related to vulnerability to capture may change. In addition, the heritable component of any physiological traits under selection could also change across environmental conditions. For example, it has been revealed that the heritability of body mass in brook charr (*S. Fontinalis*) can change drastically depending on the environment (Crespel, Bernatchez, Audet, & Garant, 2013). Similar increases in the heritability of physiological traits could accelerate the effect of selection even if the selection *per se* decreases. The heritability of physiological traits could also vary temporally. For salmonids, the heritability of traits related to body size can decrease with time, due to stronger environmental effects with age (Crespel et al., 2013; Garant, Dodson, & Bernatchez, 2003; Serbezov, Bernatchez, Olsen, & Vøllestad, 2010). If a similar change in heritability through time happens for physiological traits, this could reduce the speed of response to FIE in fisheries that target adult individuals. Some genotypes may also be more or less plastic according to the environment, increasing or reducing their vulnerability to capture. Therefore, the nature and degree of genotype‐by‐environment interactions initially present in a population are likely to accelerate or dampen any evolutionary response to fishing. Increased knowledge of the plastic, physiological responses to environmental variation would greatly contribute to our understanding or these effects.

Perhaps most importantly, by reducing the phenotypic and potentially genetic diversity across generations within targeted populations, directional selection by fishing practices could be leading wild populations into an “evolutionary trap” by making them physiologically maladapted to future environmental conditions in the absence of fishing pressure or otherwise reducing their capacity to physiologically adapt to such changes through erosion of genetic diversity. The physiological phenotypes present within a population will have a direct bearing on how they are able to respond to environmental change (Brown, Hobday, Ziegler, & Welsford, 2008; Enberg et al., 2009; Kuparinen & Hutchings, 2012; Pörtner & Farrell, 2008). Climate change, in combination with hypoxia in coastal environments due to anthropogenic pollution, is predicted to alter the geographical distribution of marine fish species, possibly due to sublethal effects on physiology (Marras et al., 2015). Selection on physiological traits by fishing could therefore lead to synergistic effects between climate change and overfishing on the abundance or distribution of species. It is also unclear whether FIE is degrading populations’ ability to rebound after fishing pressure is alleviated (Kuparinen & Hutchings, 2012), particularly in the face of environmental change. The critical lack of knowledge regarding how fish physiology and population adaptive potential are being affected by FIE may underlie this uncertainty. Reduced rates of population increase are at least partially caused by demographic shifts, reduced fecundity among individuals or altered food‐web structure, but these alone do not explain the observed lack of resilience among overexploited and collapsed fish stocks (Kuparinen & Hutchings, 2012; Marty, Dieckmann, & Ernande, 2015). It is possible that altered physiological traits could play a role, through increased natural mortality in the absence of fishing (Jørgensen & Holt, 2013; Kuparinen & Hutchings, 2012) or by contributing to the altered life‐history traits reported in exploited populations (Enberg et al., 2012).

### 3.3 Mitigation of selective effects

Attempts to alter fishing techniques to minimise harvest‐associated selection are extremely difficult to design and implement but can only be aided by a mechanistic, physiological understanding of how fish physically interact with deployed gears. There are two broad strategies for reducing harvest‐associated selection on traits within species. The first may be to restrict fishing effort to times and techniques that minimise selection. A theoretical strategy for example, may be to fish during seasons or times of day where intraspecific variation in the traits of interest, is minimised. A reduction in the within‐population variation in traits related to swim performance may be present during colder seasons or during the night when fish are inactive (Glass & Wardle, 1989). Similarly, factors such as trawl times or speeds and deployment times for traps could be altered in ways to reduce selectivity. The second general strategy is to broaden fishing effort to include a range of gears and habitat types such that selection on specific traits is diluted or countered. This could include more balanced harvesting approaches in which active and passive gears are used for the same species. Whatever the approach for minimising selection, knowing more about the physical interactions between fish and gears and the underlying physiological mechanisms will be informative for deciding which strategies to employ either alone or in combination.

A particular challenge with mitigation of within‐species selection also relates to current efforts to reduce bycatch of nontarget species. There may be a fundamental conflict in which attempts to reduce bycatch by increasing the selectivity of gears for a particular species may also increase selectivity within a species. This could additionally increase the potential for FIE to act on specific traits. For example, changes in trawl speed or position within the water column may reduce bycatch but may also cause differentiation in vulnerability within the targeted species with regard to any traits that influence vulnerability. However, increased knowledge of where the phenotypic ranges of among‐ and within‐species diversity of specific traits related to vulnerability, and particularly physiological traits, will be key in devising solutions to this apparent conflict.

## 4 FUTURE APPROACHES AND OUTSTANDING QUESTIONS

We currently know very little regarding the interplay between physiological traits and FIE. A major obstacle is that many of these questions are extremely difficult to address in an actual fisheries scenario or in the wild using free‐ranging fish. A comprehensive approach with observational and experimental work at various spatial scales, with collaboration among physiologists, behavioural ecologists, evolutionary biologists, geneticists and fishers will yield the most informative research in this field going forward.

At the smallest spatial and temporal scales, laboratory‐ and mesocosm‐based simulations of fishing procedures and selection line experiments will prove invaluable. Selection experiments have been useful for elucidating how size‐selective fisheries practices can produce an array of effects on correlated behavioural and morphological traits (Conover & Baumann, 2009; Conover & Munch, 2002; Uusi‐Heikkilä et al., 2015; Walsh, Munch, Chiba, & Conover, 2006; van Wijk et al., 2013). Selected lines also facilitate examinations of trait resiliency once selection is relaxed. To date, the majority of selection experiments have solely focused on the effects of size‐selectivity without directly considering vulnerability. In the only selection study to examine direct vulnerability to capture, Philipp and colleagues (Philipp et al., 2009) demonstrated heritability of angling vulnerability in largemouth bass and reported a range of behavioural and physiological effects associated with selection on the tendency to be captured via this method.

Further experimental approaches, such as small‐scale simulations of fishing techniques (e.g., trapping and trawling) using surrogate species, will facilitate direct exploration of links between physiology and susceptibility to capture in a manner that is not possible with full‐scale fisheries in the wild (Diaz Pauli et al., 2015; Killen, Nati et al., 2015). For example, the complete control such approaches grant us over the environment allows us to test relationships between physiological traits and vulnerability across different environmental conditions (Killen, Marras, Ryan et al., 2012; Killen, Marras, Steffensen et al., 2012; Killen et al., 2013), providing greater insight into how such mechanisms may manifest in the wild. Small‐scale fishery simulations also allow for the quantification of repeatability of vulnerability to certain gear types. A shortcoming of these experiments is that the observed trends may not extend to broader scales with other species and in more stochastic environments. However, this is always the case when examining biological phenomena in laboratory experiments. A major benefit of laboratory studies in the context of FIE is that they provide plausible and testable hypotheses that may be examined at larger scales, and inform the design of such experiments that are often expensive and logistically challenging.

At larger scales, urgently needed is information on how physiological traits may affect capture vulnerability in a natural setting (Lennox et al., 2017). Recent technological innovations in telemetry for the tracking of wild fish and remote sensing of behavioural and physiological variables (Hussey et al., 2015) are set to enable unprecedented work examining behaviour of fish around deployed gears and their accompanying physiological responses. To date, there have been few attempts to examine how laboratory‐based estimates of physiological or behavioural traits match with rates of activity in the wild (Baktoft et al., 2016), but these advances yield exciting opportunities to obtain a completely novel perspective on FIE and to understand how animals with specific physiological traits respond to fishing gears in the wild. The role of physiology in FIE presents opportunity for many new avenues of research in both laboratory and field settings, with relevant approaches encompassing telemetry, respirometry, enzyme analysis, fisheries simulations and genetics, among many others. Holistic approaches applying several of these techniques to encompass aspects of physiology, and whole organism behaviour, would be particularly powerful tools in addressing the following questions:

- Do physiological traits make some individuals more vulnerable to capture and how does this vary relative to gear type and phase of gear selectivity?
- To what degree is vulnerability to capture repeatable among individual fish?
- Does the environment modulate the intensity or direction of selection by fishing gears via effects on plasticity of physiological traits?
- Do specific physiological traits make some individuals more likely to experience mortality after escape from gear or discard?
- Does the direction or intensity of selection vary between active and passive gears?
- Does selection have long‐term effects on physiological traits and tolerance to the environment that persists even after fishing is removed?
- Do changes in mortality and life histories caused by fishing have indirect consequences for the physiological traits present within populations?
- Even if physiological traits affect vulnerability at each stage of the capture sequence, how do processes at other stages interact, counter or amplify these effects to determine overall selection on traits?
- What strategies are most effective for mitigating the physiological aspects of FIE?

## 5 CONCLUDING REMARKS

There are several avenues by which individual physiological traits may affect which fish are captured by recreational or commercial fisheries and those that are not. The influence of these traits may operate at various temporal or spatial scales, depending on the particular stage of the capture process. Selective processes may result in direct change in physiological traits associated with metabolic demand, locomotor performance, neuroendocrine function and/or sensory physiology or produce correlated responses in behavioural or life‐history traits. Conversely, in situations where selection on behavioural or morphological traits supersedes direct selection on aspects of physiology, correlated selection could still alter traits, particularly those associated with energy demand. The consequences of these effects are likely to be important for understanding synergistic effects of multiple stressors in concert with the effects of overharvest and FIE in ways that are yet to be appreciated. We hope that the possibilities raised here will encourage future work in this area.
